# Supplementary material for: Novel Mechanism for Surface Layer Shedding and Regenerating in Bacteria Exposed to Metal-Contaminated Conditions
Source: Front Microbiol. 2019 Jan 15;9:3210. doi: 10.3389/fmicb.2018.03210 (PMC6341005; doi:10.3389/fmicb.2018.03210)
Supplement: Supplementary file 1 [file Data_Sheet_1.pdf]

Supplementary Material

Novel Mechanism for Surface Layer Shedding and Regenerating in Bacteria Exposed to Metal-Contaminated Conditions

Archjana Chandramohan[1], Elodie Duprat[2], Laurent Remusat[2], Severine Zirah[1], Carine Lombard[1], Adrienne Kish[1*]

*** Correspondence:** Adrienne Kish: adrienne.kish@mnhn.fr

1 Supplementary Figures

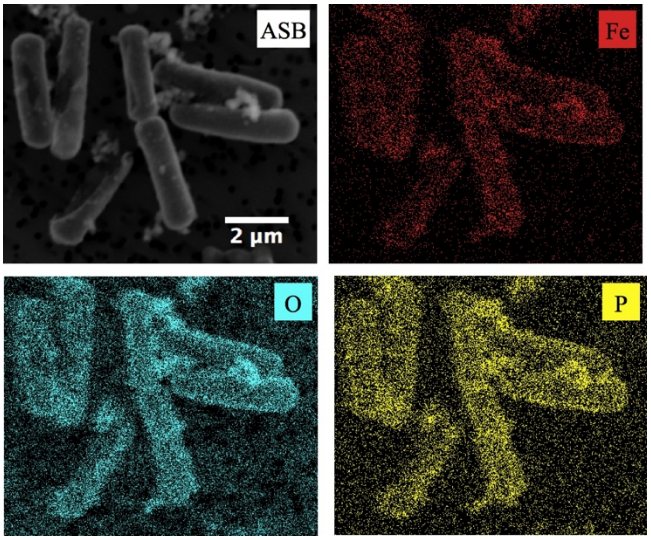

Supplementary Figure 1. SEM-EDX analysis showing Fe-phosphate mineralization on *Lysinibacillus* sp. TchIII 20n38 S-layers. Cells were examined after 5 days of recovery in rich medium after exposure to the iron-rich solution.

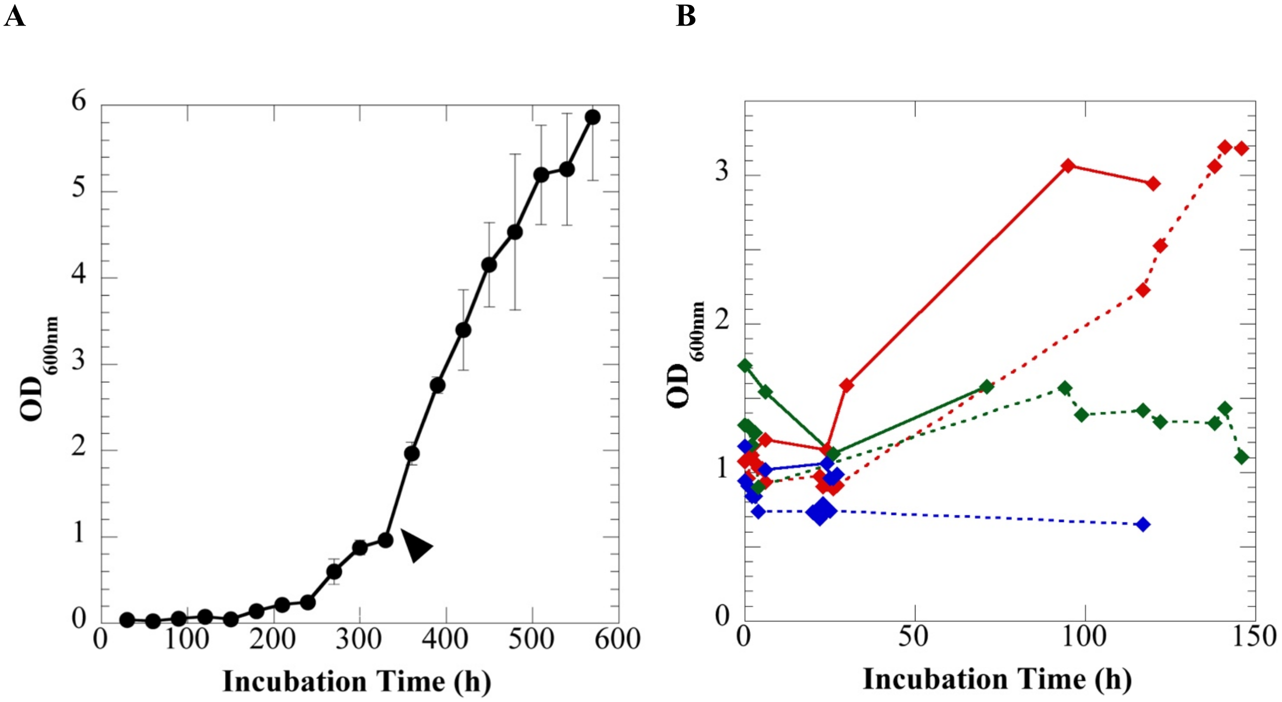

Supplementary Figure 2. Growth curves for *Lysinibacillus* sp. TchII20n38 in LB medium. (A) Growth under optimal conditions (no metal exposure). Arrowhead indicates the start of sporulation. **(B)** Growth curves of after Fe-exposure for 16h (red), 20h (blue), and 41h (green). Dotted lines indicate cultures that were in mid-exponential phase cultures (OD_{600nm} = 0.3) prior to Fe-exposure, whereas solid lines indicate cultures that were in late-exponential phase cultures (OD_{600nm} = 0.6).

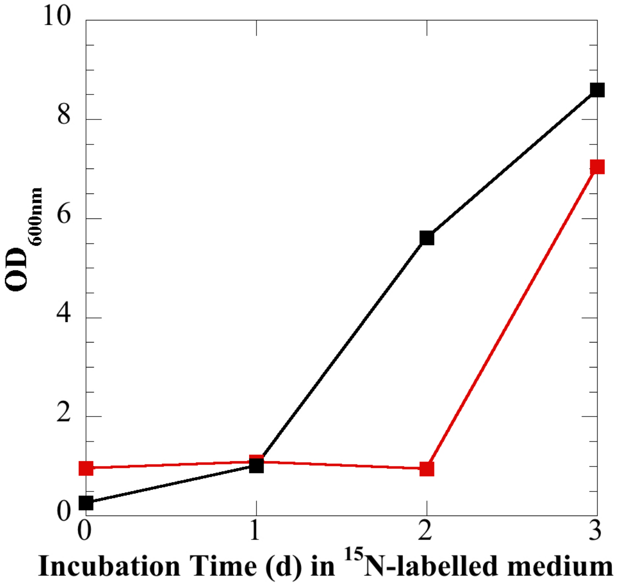

Supplementary Figure 3. Growth of *Lysinibacillus* sp. TchII20n38 cultures in ^{15}N-labelled Celtone® medium over timecourse of recovery after 16 h exposure to either a Fe-rich solution (10 mM $FeSO_4$ + 10 mM NaH_2PO_4, pH 4.5; red squares) or a buffered solution at the same pH (10 mM NaH_2PO_4, pH 4.5; black squares). Aliquots from each time-point were characterized by SEM (morphology, mineralization) and NanoSIMS (^{15}N incorporation).

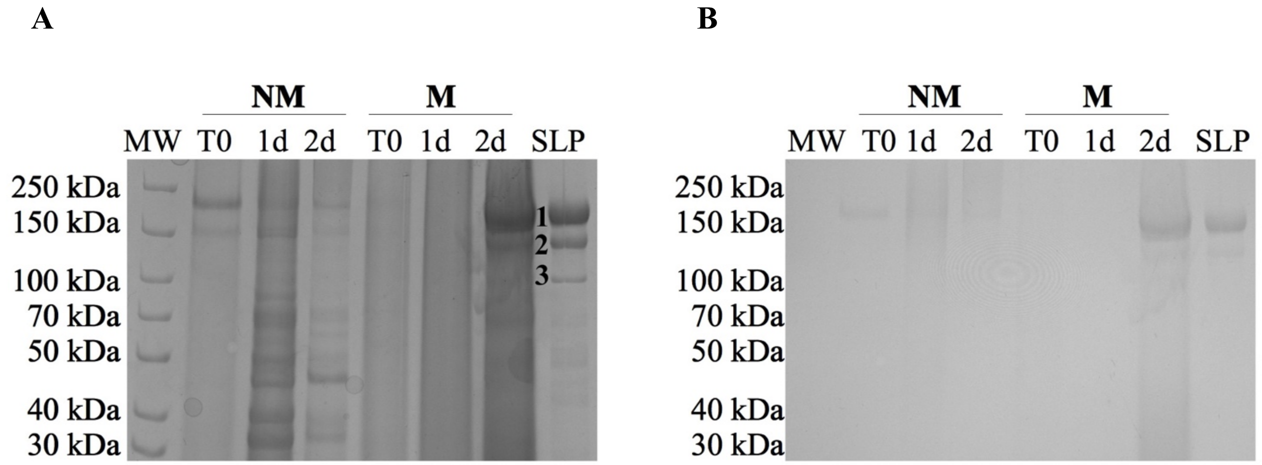

Supplementary Figure 4. SDS-PAGE of total protein from *Lysinibacillus* sp. TchII20n38 cultures over the timecourse of recovery after in ^{15}N-labelled Celtone® medium (NM = non-mineralized, M = mineralized) as well as purified S-layer protein (SLP). All samples except the purified S-layer protein controls were first treated using Tamm's reagent (see Supplementary Materials and Methods section 1.2 Characterization of S-layer Proteins) and the proteins precipitated by TCA prior to mixing with Laemmli buffer. In addition to Coomassie blue staining for total protein (**panel A**), a duplicate gel was stained with periodic acid-Schiff's reagent (PAS) (**panel B**) to detect the post-translational glycosylation characteristic of *Lysinibacillus* S-layer proteins. The three bands (indicated by the bold numbers beside the bands excised for both samples M_2d and SLP) correspond to the purified S-layer protein sample were analyzed by ultra-high performance liquid chromatography - mass spectrometry (UHPLC-MS).

NM_2d_Band 1

```
        10         20         30         40         50         60         70         80         90        100
MANQPKKYKK FVATAATATL VAAAIVPVAS AASFTDVTDN NSHKEAISNL VDAGIIKGYP DGTFKPDQYV TRGQVVKLLG RWLETEGYEI PTDWNSKQRF
       110        120        130        140        150        160        170        180        190        200
NDLPVTTADK ELVQYAALAK DAGAFNGVNG NLNYSQNMAR QQMAIVLVRA IKEIQEVDLV KEYKDAKFTS EISDLGGLNT EQREAITALE YAELTKAATL
       210        220        230        240        250        260        270        280        290        300
PGKAFKPNDT ITRAQFASFL DRTMKLEFTA PAKAAVKAIN NTTVEITFEQ EVENVQALNF EIKDLEIKNA AVKQTNKKVV VLTTASQTAD KEYTVSLDAE
       310        320        330        340        350        360        370        380        390        400
EIGKFKGVSA VVPTKVELVA PSFQGKLGQQ VTVKAQVTVA EGQSKAGIPV TFYVPGNSND GVTPTVTGEA ITDENGVASY SYTRYKDGVD EVTAYATGDR
       410        420        430        440        450        460        470        480        490        500
SKFSLGYVFW GVDTILSIEE VTKDATINNG ANKTYKVTYK DPKTGKPEAN KTFNVGFVEN MNVTSDKVAN ATVNGVKALQ LSNATVLEAA QITTDSKGEA
       510        520        530        540        550        560        570        580        590        600
TFTVSGTNAT VTPVVYDLHD TKNNVSNKKY SASALQATAS KVNFAAVQAD YTLEMTRTGK EVAAIGETNG REYKIVVKDK EGKLAKNEVV NVAFNEDLDR
       610        620        630        640        650        660        670        680        690        700
VISTVTNAKF IDDENEVYYT GNEAKQISVK TDEKGEATFV IGSDKINDYA TPIAWIDINT SNAKQGTLDE GEPKVVGQMS YFQEAYLDGA SIKSYLNAKK
       710        720        730        740        750        760        770        780        790        800
ETKFKDNEIA TFKAQLVNQS DKVISASIKS VSYTVYNTGS NDIEVAGKKV SPNRSITLSY PGELSSADLD VKAVDGKSTS VKVIATGVAI NPKDGKDYAF
       810        820        830        840        850        860        870        880        890        900
TAKEATATFT STSEVPSNFI GRVESVNTSD KTLTFVGKKP VSLKDAKFVA ENNAELSDLD AFVEELKKYT SGVEVQYIKN SEGKVTFKIV RAAIGGGSGV
       910        920        930        940        950        960        970        980        990       1000
TVDTYVTTLD SAKIKVAGST TTLATTTIDS AQLLTTGNTL EVDGVKFEQH RGATPTDLYY ADLADLANKI NKNTRIKIQA KVVSGILELT DTRTGKFEVI
      1010       1020       1030       1040       1050       1060       1070       1080       1090       1100
LNGNKVESSA GTVGVAQEIT FNFTSSFNVA SGDKVLVNGT EGTVASVSSD KKTVVVKFTS TLIPTGTSIS TFTVKDKETS NTITINPSGV NIQP
```

NM_2d_Band 2

```
        10         20         30         40         50         60         70         80         90        100
MANQPKKYKK FVATAATATL VAAAIVPVAS AASFTDVTDN NSHKEAISNL VDAGIIKGYP DGTFKPDQYV TRGQVVKLLG RWLETEGYEI PTDWNSKQRF
       110        120        130        140        150        160        170        180        190        200
NDLPVTTADK ELVQYAALAK DAGAFNGVNG NLNYSQNMAR QQMAIVLVRA IKEIQEVDLV KEYKDAKFTS EISDLGGLNT EQREAITALE YAELTKAATL
       210        220        230        240        250        260        270        280        290        300
PGKAFKPNDT ITRAQFASFL DRTMKLEFTA PAKAAVKAIN NTTVEITFEQ EVENVQALNF EIKDLEIKNA AVKQTNKKVV VLTTASQTAD KEYTVSLDAE
       310        320        330        340        350        360        370        380        390        400
EIGKFKGVSA VVPTKVELVA PSFQGKLGQQ VTVKAQVTVA EGQSKAGIPV TFYVPGNSND GVTPTVTGEA ITDENGVASY SYTRYKDGVD EVTAYATGDR
       410        420        430        440        450        460        470        480        490        500
SKFSLGYVFW GVDTILSIEE VTKDATINNG ANKTYKVTYK DPKTGKPEAN KTFNVGFVEN MNVTSDKVAN ATVNGVKALQ LSNATVLEAA QITTDSKGEA
       510        520        530        540        550        560        570        580        590        600
TFTVSGTNAT VTPVVYDLHD TKNNVSNKKY SASALQATAS KVNFAAVQAD YTLEMTRTGK EVAAIGETNG REYKIVVKDK EGKLAKNEVV NVAFNEDLDR
       610        620        630        640        650        660        670        680        690        700
VISTVTNAKF IDDENEVYYT GNEAKQISVK TDEKGEATFV IGSDKINDYA TPIAWIDINT SNAKQGTLDE GEPKVVGQMS YFQEAYLDGA SIKSYLNAKK
       710        720        730        740        750        760        770        780        790        800
ETKFKDNEIA TFKAQLVNQS DKVISASIKS VSYTVYNTGS NDIEVAGKKV SPNRSITLSY PGELSSADLD VKAVDGKSTS VKVIATGVAI NPKDGKDYAF
       810        820        830        840        850        860        870        880        890        900
TAKEATATFT STSEVPSNFI GRVESVNTSD KTLTFVGKKP VSLKDAKFVA ENNAELSDLD AFVEELKKYT SGVEVQYIKN SEGKVTFKIV RAAIGGGSGV
       910        920        930        940        950        960        970        980        990       1000
TVDTYVTTLD SAKIKVAGST TTLATTTIDS AQLLTTGNTL EVDGVKFEQH RGATPTDLYY ADLADLANKI NKNTRIKIQA KVVSGILELT DTRTGKFEVI
      1010       1020       1030       1040       1050       1060       1070       1080       1090       1100
LNGNKVESSA GTVGVAQEIT FNFTSSFNVA SGDKVLVNGT EGTVASVSSD KKTVVVKFTS TLIPTGTSIS TFTVKDKETS NTITINPSGV NIQP
```

Supplementary Material

NM_2d_Band 3

```
10         20         30         40         50         60         70         80         90         100
MANQPKKYKK FVATAATATL VAAAIVPVAS AASFTDVTDN NSHKEAISNL VDAGIIKGYP DGTFKPDQYV TRGQVVKLLG RWLETEGYEI PTDWNSKQRF
110        120        130        140        150        160        170        180        190        200
NDLPVTTADK ELVQYAALAK DAGAFNGVNG NLNYSQNMAR QQMAIVLVRA IKEIQEVDLV KEYKDAKFTS EISDLGGLNT EQREAITALE YAELTKAATL
210        220        230        240        250        260        270        280        290        300
PGKAFKPNDT ITRAQFASFL DRTMKLEFTA PAKAAVKAIN NTTVEITFEQ EVENVQALNF EIKDLEIKNA AVKQTNKKVV VLTTASQTAD KEYTVSLDAE
310        320        330        340        350        360        370        380        390        400
EIGKFKGVSA VVPTKVELVA PSFQGKLGQQ VTVKAQVTVA EGQSKAGIPV TFYVPGNSND GVTPTVTGEA ITDENGVASY SYTRYKDGVD EVTAYATGDR
410        420        430        440        450        460        470        480        490        500
SKFSLGYVFW GVDTILSIEE VTKDATINNG ANKTYKVTYK DPKTGKPEAN KTFNVGFVEN MNVTSDKVAN ATVNGVKALQ LSNATVLEAA QITTDSKGEA
510        520        530        540        550        560        570        580        590        600
TFTVSGTNAT VTPVVYDLHD TKNNVSNKKY SASALQATAS KVNFAAVQAD YTLEMTRTGK EVAAIGETNG REYKIVVKDK EGKLAKNEVV NVAFNEDLDR
610        620        630        640        650        660        670        680        690        700
VISTVTNAKF IDDENEVYYT GNEAKQISVK TDEKGEATFV IGSDKINDYA TPIAWIDINT SNAKQGTLDE GEPKVVGQMS YFQEAYLDGA SIKSYLNAKK
710        720        730        740        750        760        770        780        790        800
ETKFKDNEIA TFKAQLVNQS DKVISASIKS VSYTVYNTGS NDIEVAGKKV SPNRSITLSY PGELSSADLD VKAVDGKSTS VKVIATGVAI NPKDGKDYAF
810        820        830        840        850        860        870        880        890        900
TAKEATATFT STSEVPSNFI GRVESVNTSD KTLTFVGKKP VSLKDAKFVA ENNAELSDLD AFVEELKKYT SGVEVQYIKN SEGKVTFKIV RAAIGGGSGV
910        920        930        940        950        960        970        980        990        1000
TVDTYVTTLD SAKIKVAGST TTLATTTIDS AQLLTTGNTL EVDGVKFEQH RGATPTDLYY ADLADLANKI NKNTRIKIQA KVVSGILELT DTRTGKFEVI
1010       1020       1030       1040       1050       1060       1070       1080       1090       1100
LNGNKVESSA GTVGVAQEIT FNFTSSFNVA SGDKVLVNGT EGTVASVSSD KKTVVVKFTS TLIPTGTSIS TFTVKDKETS NTITINPSGV NIQP
```

M_2d_Band 1

```
10         20         30         40         50         60         70         80         90         100
MANQPKKYKK FVATAATATL VAAAIVPVAS AASFTDVTDN NSHKEAISNL VDAGIIKGYP DGTFKPDQYV TRGQVVKLLG RWLETEGYEI PTDWNSKQRF
110        120        130        140        150        160        170        180        190        200
NDLPVTTADK ELVQYAALAK DAGAFNGVNG NLNYSQNMAR QQMAIVLVRA IKEIQEVDLV KEYKDAKFTS EISDLGGLNT EQREAITALE YAELTKAATL
210        220        230        240        250        260        270        280        290        300
PGKAFKPNDT ITRAQFASFL DRTMKLEFTA PAKAAVKAIN NTTVEITFEQ EVENVQALNF EIKDLEIKNA AVKQTNKKVV VLTTASQTAD KEYTVSLDAE
310        320        330        340        350        360        370        380        390        400
EIGKFKGVSA VVPTKVELVA PSFQGKLGQQ VTVKAQVTVA EGQSKAGIPV TFYVPGNSND GVTPTVTGEA ITDENGVASY SYTRYKDGVD EVTAYATGDR
410        420        430        440        450        460        470        480        490        500
SKFSLGYVFW GVDTILSIEE VTKDATINNG ANKTYKVTYK DPKTGKPEAN KTFNVGFVEN MNVTSDKVAN ATVNGVKALQ LSNATVLEAA QITTDSKGEA
510        520        530        540        550        560        570        580        590        600
TFTVSGTNAT VTPVVYDLHD TKNNVSNKKY SASALQATAS KVNFAAVQAD YTLEMTRTGK EVAAIGETNG REYKIVVKDK EGKLAKNEVV NVAFNEDLDR
610        620        630        640        650        660        670        680        690        700
VISTVTNAKF IDDENEVYYT GNEAKQISVK TDEKGEATFV IGSDKINDYA TPIAWIDINT SNAKQGTLDE GEPKVVGQMS YFQEAYLDGA SIKSYLNAKK
710        720        730        740        750        760        770        780        790        800
ETKFKDNEIA TFKAQLVNQS DKVISASIKS VSYTVYNTGS NDIEVAGKKV SPNRSITLSY PGELSSADLD VKAVDGKSTS VKVIATGVAI NPKDGKDYAF
810        820        830        840        850        860        870        880        890        900
TAKEATATFT STSEVPSNFI GRVESVNTSD KTLTFVGKKP VSLKDAKFVA ENNAELSDLD AFVEELKKYT SGVEVQYIKN SEGKVTFKIV RAAIGGGSGV
910        920        930        940        950        960        970        980        990        1000
TVDTYVTTLD SAKIKVAGST TTLATTTIDS AQLLTTGNTL EVDGVKFEQH RGATPTDLYY ADLADLANKI NKNTRIKIQA KVVSGILELT DTRTGKFEVI
1010       1020       1030       1040       1050       1060       1070       1080       1090       1100
LNGNKVESSA GTVGVAQEIT FNFTSSFNVA SGDKVLVNGT EGTVASVSSD KKTVVVKFTS TLIPTGTSIS TFTVKDKETS NTITINPSGV NIQP
```

M_2d_Band 2

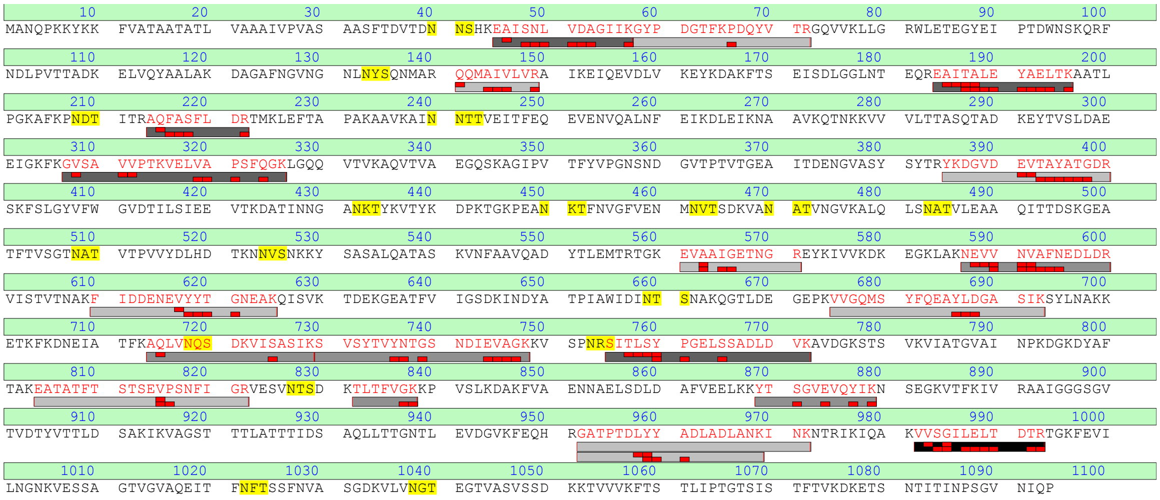

M_2d_Band 3

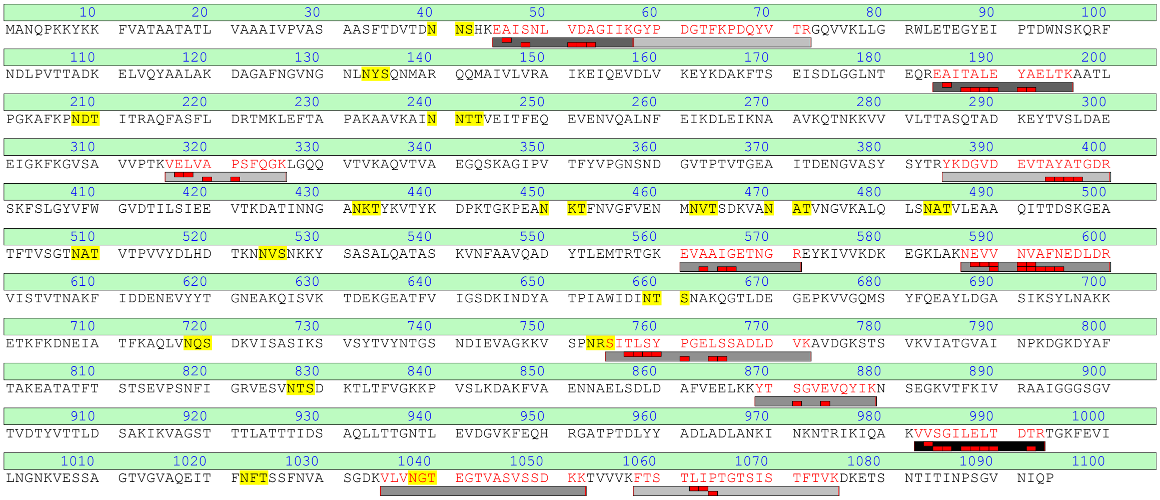

Supplementary Figure 5. Identification of S-layer protein by mass spectrometry from samples after 2 d of recovery after Fe-mineralization (NM_2d and M_2d for non-mineralized and mineralized samples, respectively). Analyses of all three bands analyzed for each sample are shown (see Supplementary Figure 4). Identified sequence is shown in red. The rectangles below the sequence correspond to the detected peptides, with colors from light grey to black with increasing scores, and red boxes showing the b- and y-type ions detected on the MS/MS spectra (Figure generated with ProteinScape, Bruker Daltonics). The list of peptides for samples SLP (purified S-layer protein) and M_2d are available (see supplementary dataset files for **Table S3** and **Table S4**, respectively).

Supplementary Material

NM_T0_rep1

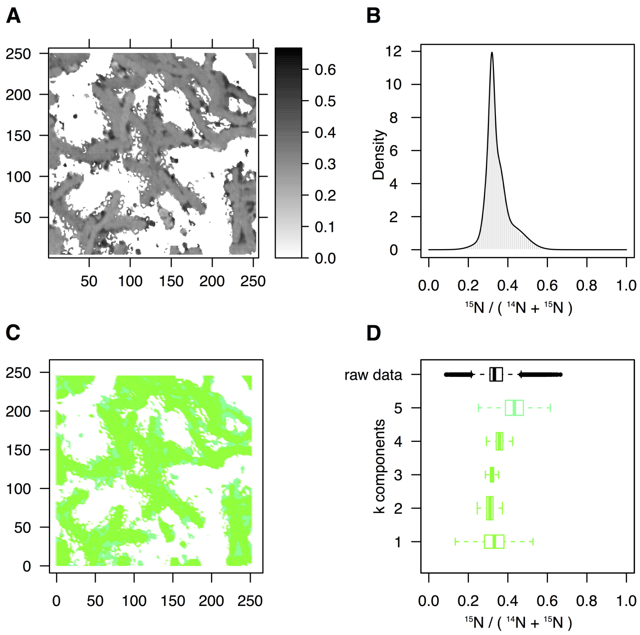

NM_1d_rep1

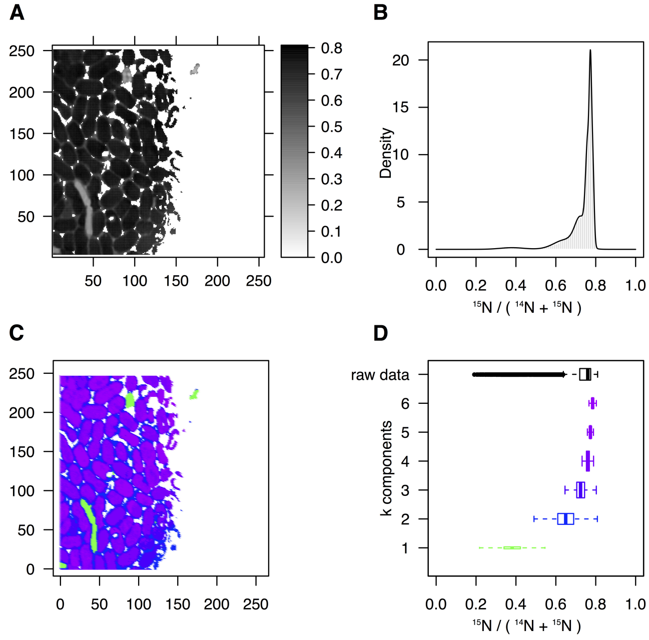

NM_2d_rep1

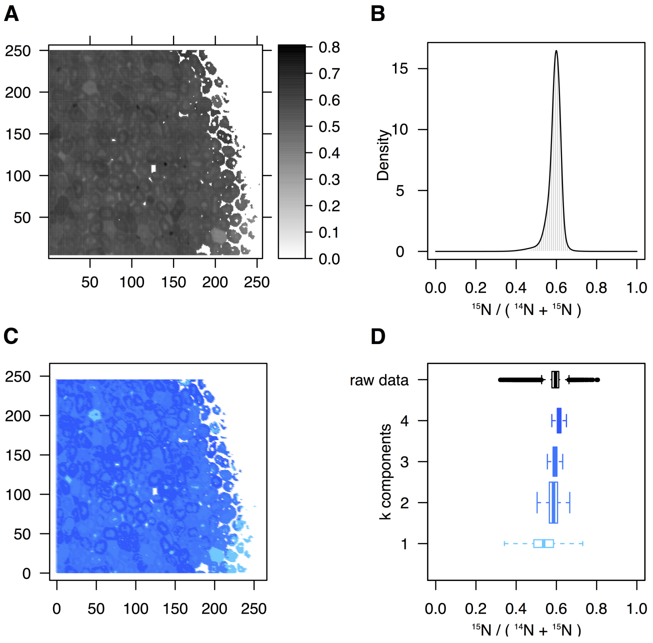

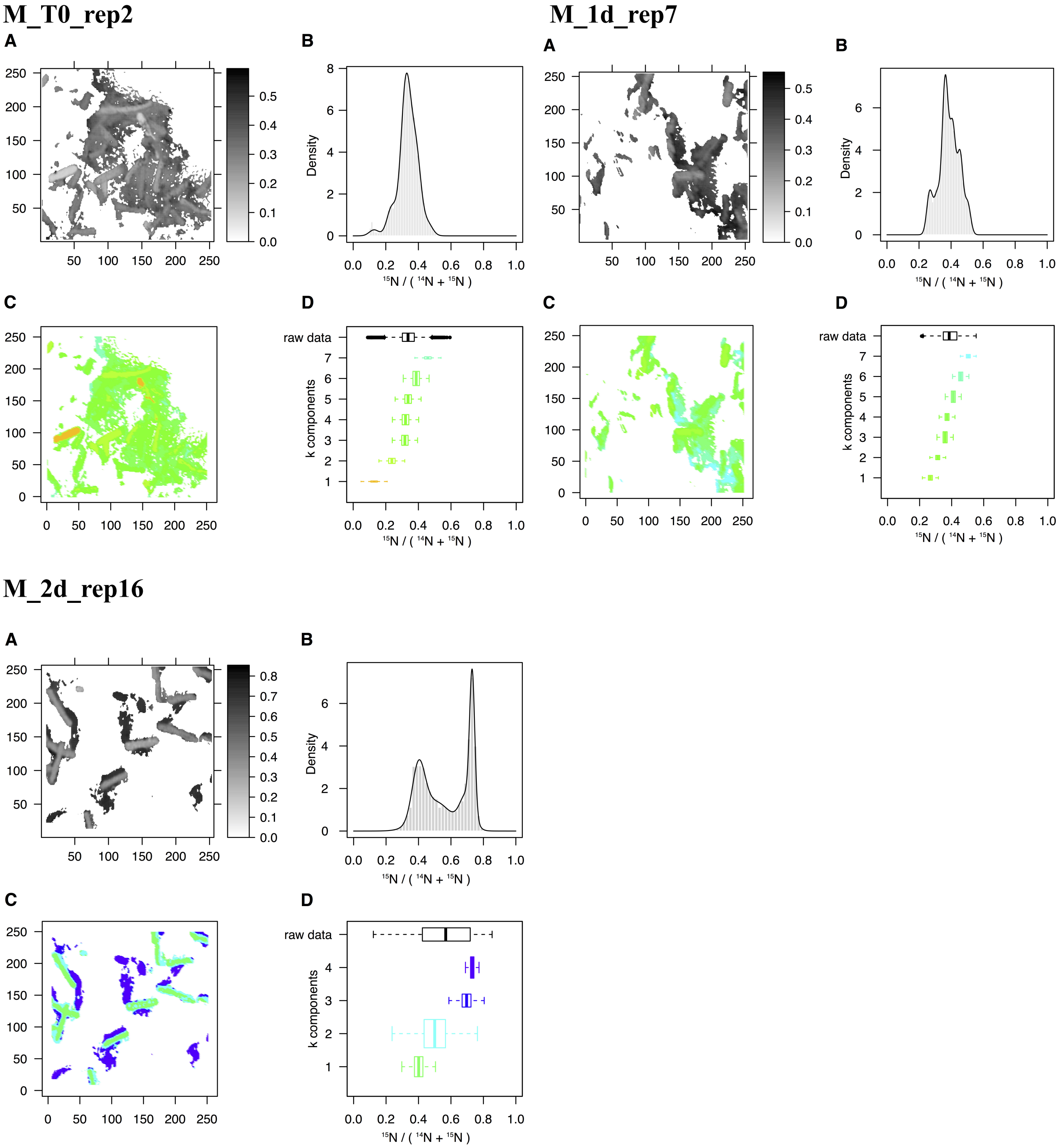

Supplementary Figure 6. Statistical analysis of ^{15}N/(^{14}N+^{15}N) NanoSIMS image for all time course samples. **(A)** Processed ^{15}N/(^{14}N+^{15}N) ratio map. **(B)** Processed ^{15}N/(^{14}N+^{15}N) ratio histogram (gray bars) and probability density (dark line) estimated by Gaussian mixture modeling. **(C)** Map of the pixel subpopulations (see (D) for color code). **(D)** Decomposition of the distribution of processed ^{15}N/(^{14}N+^{15}N) ratio into 4 Gaussian components of unequal variance (NM_T0_rep1: unequal variance with 5 components; NM_1d_rep1: unequal variance with 6 components; NM_2d_rep1: unequal variance with 4 components; M_T0_rep2: equal variance with 7 components; M_1d_rep7: equal variance with 7 components). For sample M_2d_rep16, see **Fig. 6** in the main text.

2 Supplementary Materials and Methods

2.1 Sequencing of the S-layer gene by genome walking

The sequence of the gene encoding the S-layer protein of *Lysinibacillus* sp. TchIII 20n38 was obtained by genome walking, based on successive steps of digestion, ligation and PCR. The first primers were designed based on the conserved sequences encoding the S-layer homology (SLH) domains of S-layer proteins (Lederer et al., 2013). The list of primers is provided in Table S1. Genomic DNA was extracted from 10 mL bacterial culture in LB medium, using a classical lysozyme-EDTA/SDS-proteinase K treatment, followed by chloroform-isopropanol extraction (Moore et al., 2004). DNA was digested with the restriction enzymes EcoRI or HindIII (New England Biolabs), and ligated with DNA ligase T4 (Promega). The PCRs were performed using KOD DNA polymerase (Novagen) in a Veriti (Applied Biosystems) or GenePro (Bioer) thermocycler, using the following sequence: 2 min at 95 °C followed by 35 cycles (20 s at 95°C, 10 s between 53 and 58 °C, 1 min at 70 °C), and 5 min at 70 °C. The PCR products were sequenced by Eurofins MWG Operon. The gene and protein sequences were deposited in Genbank (accession number MH879151).

Table S1. Primers used in this study.

Name	Sequence
1-F	TAGCTTCTGCTATCGTACCAGTG
1-R	TGAACCAATTACAAACGTAGCTTCACC
2-F	TGTTACTCGTGGTCAAGTGGTTAAA
2-R	GCCATCTGGATATCCTTTGATGATAC
3-F	CTTGACGCTGAAGAAATCGGTAAAT
3-R	ACTCTTTGTCAGCTGTTTGAGAAGC
4-F	ACCAACAGACCTTTACTATGCAGAT
4-R	TGTTCGAATTTAACACCATCTACTTC

2.2 Characterization of S-layer proteins

Bacterial cell pellets were obtained throughout the time course of recovery (T0, 1 d, 2 d) following Fe-mineralization (see section 2.2 "Mineralization Recovery Time Course" in the main text) by gentle centrifugation (2600 x g, 15 min followed by flash freezing in liquid nitrogen and storage at -80 °C until sample processing. All time course samples were subjected to a demineralization treatment prior to mass spectrometry analyses by adding 840 µL of Tamm's reagent (0.1 M oxalic acid, 0.0175 M ammonium oxalate) to each cell pellet, followed by protein precipitation using 160 µl TCA (6.1 N). After 10 minutes stirring in the dark, the samples were centrifuged (18000 x g, 30 min) and resuspended in 60 µL of Laemmli buffer. The extracted S-layers were submitted to sodium dodecyl sulfate-polyacrylamide gel electrophoresis (SDS-PAGE) and the bands of interest were digested with trypsin, as described previously (François et al., 2012). The S-layer digests were analyzed by ultra-high performance liquid chromatography - mass spectrometry (UHPLC-MS) on an Ultimate 3000 RSLC chromatographic system (Thermo Scientific) connected to a high-resolution electrospray ionization – quadrupole – time of flight (ESI-Q-TOF) mass spectrometer (Maxis II ETD, Bruker Daltonics). The separation was achieved on an Acclaim RSLC Polar Advantage II column (2.2 µm, 2.1 × 100 mm, Thermo Scientific) at a flow rate of 300 µL/min, using the following gradient of solvent A (milliQ water / 0.1% formic acid) and solvent B (HPLC-MS grade acetonitrile / 0.08% formic acid) over a total run time of 17.5 min: linear increase from 10% B to 60% B for 12 min, linear increase to 100% B for 0.2 min, decrease to 10% B for 0.5 min. The ESI-Q-TOF instrument was externally calibrated before each run using a sodium formate solution consisting of 10 mM sodium hydroxide in isopropanol / 0.2% formic acid (1:1, v/v). The MS spectra were acquired in positive ion mode in the mass range m/z 60 – 2000. The source parameters were as follows: nebulizer gas 35 psi, dry gas 8 L/min, capillary voltage 3500 V, end plate offset 500 V, temperature 200 °C. Automatic MS/MS was carried out using the following set-up: absolute threshold 500 counts, preferred charge states: 1–3, unknown charge states excluded, cycle time 3 s, MS spectra rate: 2 Hz, MS/MS spectra rate: 3 Hz at 5000 counts increasing to 6 Hz at 50 000 counts or above. MS/MS active exclusion was set after 1 spectrum unless intensity increased fivefold. Collision energy was automatically calculated from *m/z* and charge states. The LC-MS/MS data were treated with Data Analysis 4.3 and ProteinScape (Bruker Daltonics).

Table S2. Identification of the S-layer protein in non-mineralized and mineralized samples, based on bottom-up proteomics. Number of identified peptides and protein sequence coverage are indicated for each of the three bands containing peptides assigned to the S-layer protein (see Supplementary Figure 4 for details).

	# Peptides	% Coverage
**Band 1**		
SLP	100	80.4
M_2d	18	24.3
**Band 2**		
SLP	93	84.4
M_2d	20	25.4
**Band 3**		
SLP	92	84.0
M_2d	12	15.5

3 References

François, F., Lombard, C., Guigner, J.-M., Soreau, P., Brian-Jaisson, F., Martino, G., et al. (2012). Isolation and characterization of environmental bacteria capable of extracellular biosorption of mercury. *Appl Environ Microbiol* 78, 1097–1106. doi:10.1128/AEM.06522-11.

Lederer, F. L., Weinert, U., Günther, T. J., Raff, J., Raff, J., Weiß, S., et al. (2013). Identification of multiple putative S-layer genes partly expressed by *Lysinibacillus sphaericus* JG-B53. *Microbiology* 159, 1097–1108. doi:10.1099/mic.0.065763-0.

Moore, E., Arnscheidt, A., Krüger, A., Strompl, C., and Mau, M. (2004). "Section 1 - Isolation of Nucleic Acids," in *Molecular Microbial Ecology Manual*, eds.G. A. Kowalchuk, F. J. de Bruijn, I. M. Head, A. D. Akkermans, and J. D. van Elsas (Dordrecht: Springer, Dordrecht), 1–212. doi:10.1007/978-1-4020-2177-0_1.
